# Sonocrystallization of a novel ZIF/zeolite composite adsorbent with high chemical stability for removal of the pharmaceutical pollutant azithromycin from contaminated water

**DOI:** 10.1016/j.ultsonch.2023.106463

**Published:** 2023-05-30

**Authors:** Zhiming Liu, Ashkan Bahadoran, As'ad Alizadeh, Nafiseh Emami, Tariq J. Al-Musaw, Ahmed Hussien Radie Alawadi, Aseel M. Aljeboree, Mahmoud Shamsborhan, Iman Najafipour, Seyed Erfan Mousavi, Milad Mosallanezhad, Davood Toghraie

**Affiliations:** a RENMIN Hospital of Wuhan University, Department of Stomatology, Wuhan, Hubei 430060, China; b Materials Interfaces Center, Shenzhen Institutes of Advanced Technology, Chinese Academy of Sciences, Shenzhen 518055, Guangdong, China; c Department of Civil Engineering, College of Engineering, Cihan University-Erbil, Erbil, Iraq; d Department of Chemical Engineering, Faculty of Engineering, University of Isfahan, Isfahan, Iran; e Building and Construction Techniques Engineering Department, Al-Mustaqbal University College, 51001 Hillah, Babylon, Iraq; f College of Medical Technology Techniques, The Islamic University, Najaf, Iraq; g Department of Chemistry, College of Science for Women, University of Babylon, Hilla, Iraq; h Department of Mechanical Engineering, College of Engineering, University of Zakho, Zakho, Iraq; i Department of Chemical Engineering, College of Engineering, University of Tehran, Tehran, Iran; j Department of Mechanical Engineering, Khomeinishahr Branch, Islamic Azad University, Khomeinishahr Khomeinishahr, Iran

**Keywords:** Azithromycin, Pharmaceutical Contaminant, Adsorption, ZIF-8/Zeolite composite, Langmuir isotherm

## Abstract

Water pollution management, reduction, and elimination are critical challenges of the current era that threaten millions of lives. By spreading the coronavirus in December 2019, the use of antibiotics, such as azithromycin increased. This drug was not metabolized, and entered the surface waters. ZIF-8/Zeolit composite was made by the sonochemical method. Furthermore, the effect of pH, the regeneration of adsorbents, kinetics, isotherms, and thermodynamics were attended. The adsorption capacity of zeolite, ZIF-8, and the composite ZIF-8/Zeolite were 22.37, 235.3, and 131 mg/g, respectively. The adsorbent reaches the equilibrium in 60 min, and at pH = 8. The adsorption process was spontaneous, endothermic associated with increased entropy. The results of the experiment were analyzed using Langmuir isotherms and pseudo-second order kinetic models with a R2 of 0.99, and successfully removing the composite by 85% in 10 cycles. It indicated that the maximum amount of drug could be removed with a small amount of composite.

## Highlights

• A novel Zeolite/ZIF-8 composite was synthesized by Sonocrystallization method.
• The adsorption capacity of the composite ZIF-8/Zeolite was 131 mg/g, respectively.
• Investigation of effect of pH and electrostatic interaction mechanism on the Zeolite/ZIF-8 composite.
• The adsorbent reaches equilibrium in 60 min at pH = 8.
• The recovery of adsorbent show that the removal efficiency was 85%.

## Graphical abstract

**Figure:**
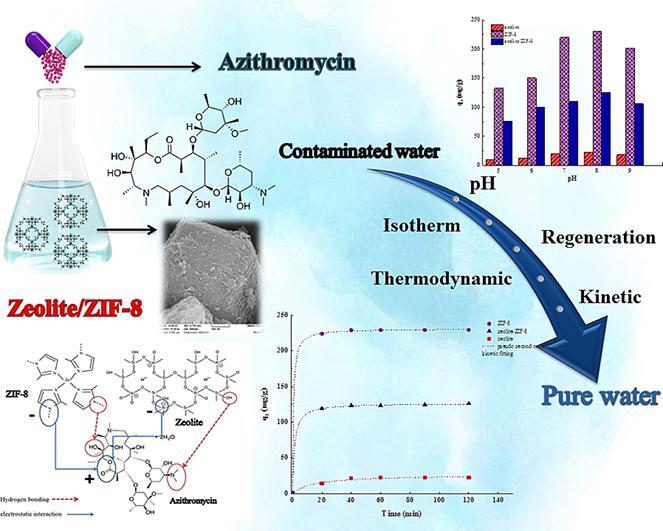

## 1 Introduction

The water crisis is the foremost vital topic in today's world. Nowadays, the scarcity of clean water resources, and pollution have seriously endangered people's lives, especially in developing countries [1], [2], [3]. Diseases caused by polluted water are the reason of many deaths in developing countries [4], [5], [6]. The water scarcity crisis in the last century had more casualties than infectious diseases and HIV. Therefore, the lack of clean water resources led to the wastewater treatment [7], [8]. There were sustainable organic compounds, such as the personal care products, and medicines in municipal wastewater, natural waters, and even drinking water in the last decade. These drugs can enter water resources via various routes, such as municipal sewage, hospitals, veterinary, and farms [9], [10]. By coronavirus outbreak in 2019, a broad proceeding was taken to decrease the transmission of SARS-CoV-2 [11], [12]. Early in the pandemic, it was proposed to use the antibiotics to treat acute and severe cases of Covid-19, and by spreading the virus, large quantities of antibiotics were prescribed [13], [14], [15]. Azithromycin is one of the most widely used antibiotics. It is an antibiotic used to prevent the infections caused by certain bacteria [16]. Prescribing antibiotics should not be underestimated because of continued utilization of antibiotics, and their entry into the water cycle lead to drug resistance [17], [18]. As drug resistance increases in living organisms, common infections that can be easily treated may become more difficult for treating. The biggest problem is the increase in the multidrug-resistant bacteria [19]. Researchers used the simulation methods to adsorb drugs [20], [21]. Various methods, such as membrane, adsorption, ozonation, and advanced oxidation processes (AOP) were used to remove the medicinal compounds from water [22], [23], [24], [25]. The materials, such as activated carbon, metal–organic frameworks (MOFs), and polymeric adsorbents have a high particular surface area, and permeable structure, and are modifiable, which can be compelled in specific execution, and expulsion of contaminants [26]. MOFs showed high ability in the adsorption and loading of materials. They are made from the coordination between metal clusters and organic ligands. MOFs appear to moderately have small instability than ordinary porous materials [27]. Using MOFs in the environmental applications attracted attention in recent years [28], [29], [30], [31], [32], [33], [34]. ZIF-8 was used for the adsorption of Norfloxacin in polluted water. The maximum adsorption measured at 40 °C, pH 5, and C_0_ = 10–70 mg/l, was 69.4 mg/g for Norfloxacin which is poorly functional compared to other MOFs due to the effect of adsorption mechanisms [35]. One of the bugs associated using MOFs was their regeneration and the chemical and thermal stability of these materials in an aqueous environment. The mechanism of instability against water is caused by two reactions of ligand displacement and hydrolysis.

In the process of ligand displacement, the cation (metal) binds with water and the ligand is released. In Hydrolysis, the metal–ligand bond is broken, and the water molecule, which is decomposed into hydroxyl ion, enters the bond with the metal and the ligand [36].

The key factor in the stability of MOFs is that the metal cluster is neutral. Otherwise, the electrophilic metal enters the bond with oxygen and reduces the porosity. In addition to the neutrality of the metal cluster, the metal–ligand bond strength is an important parameter in stability. The hydrophobicity of the ligand and the metal plays an important role in the stability of the MOF, because it does not have a tendency to coordinate with water [37], [38].

The stability period of MOFs is calculated as follow. A certain amount of MOF is poured into a certain amount of water at room temperature and stirred, and periodically, the MOF is separated from the aqueous environment and dried, and an XRD test is taken from it to check its crystallinity [18]. The stability for ZIF-8 in water at 100 ^°^C were reported to be seven days and in water at room temperature is one month. The measured stability period was low compared to the zeolites and UiO-66 which is equal to 1 month. Moreover, measured stability period was high compared to HKUST-1 which is equal to 1 day [39]. The properties of MOFs can be improved by combining MOFs with the zeolites, carbon, and polymeric materials [40], [41], [42], [43]. Composite materials consisting of MOF were one of the suitable method to increase the adsorption loading [44], [45], [46], [47], [48], [49]. For example, Ghiasi et al. [22] investigated the adsorption of diphenhydramin by MIL101-OH/Chitosan. The results showed that by adding polymer to MOF, stability and adsorption capacity increase. Xing et al. [15] investigated the adsorption of Doxycycline and Naproxen by HKUST-1/ZnO/SA. The results showed that polymers and nanoparticles decrease the specific surface area but improve the selective adsorption and stability of the MOF. Sonocrystallization is the crystallization induced by ultrasound, and was first reported by Richards and Loomis in 1927 [50]. Sonocrystallization involves applying the ultrasound energy to control the nucleation, and crystal growth of a crystallization process [51]. When ultrasound is applied to a solution for crystallization, it can affect the properties of crystalline products significantly [52], [53]. Extensive research was done for synthesis and the sonocrystallization of MOFs [54], [55], [56]. For example, Seoane et al. [57] investigated the synthesis of ZIF-8, ZIF-11 and ZIF-20 by sonocrystallization method. Zheng et al. [58] investigated the sonocrystalization of ZIF-8/ZnO. They used ultrasonic waves to form MOF crystals. In terms of using the ultrasound waves for the formation of MOF crystals, the title of sonocrystallization was chosen.

Due to the importance of accurate, healthy and cost-effective synthesis of nanostructured adsorbents, an easy, green and energy-free method at room temperature called green synthesis of MOFs has been noticed in the last few decades. Important parameters for the synthesis of MOFs are the selection of inorganic metal cations, organic ligand molecules, used solvent and operating conditions including temperature, pressure and a suitable reactor [59].

The solutions that are important for green synthesis are [59]:

• To use and produce materials, the synthesis method must be designed in such a way that it is non-toxic or low-toxic for human health and the environment. This means that solvents that break down into dangerous products during synthesis should preferably not be used. For example, the solvent dimethylformamide is not only a dangerous chemical substance, but it quickly turns into dimethylamine upon hydrolysis. Water solvent and organic solvents produced from renewable feedstock such as ethanol can be used instead.
• The energy required for synthesis should be economically viable. Synthetic methods should be carried out at ambient pressure and temperature. Reducing energy consumption can be achieved mainly by synthesis at temperatures as low as possible, using alternative energy methods such as ultrasound or mechanochemistry.
• Chemical products should be designed in such a way that at the end of the operation, they become harmless products in the environment. Also, preferably, the substance used in a chemical process should be chosen so that chemical accidents, including explosions and fires, are minimized. This issue is especially important for large-scale synthesis, which is ignored.
• In general, it can be concluded that in any case, the amount of solvent used for the synthesis and activation or purification of MOF should be as low as possible, and also optimizing the reaction time is very important to reduce energy consumption.

The best desired solvent for the synthesis of MOFs is water and ethanol due to its harmless properties and existing technologies for purification and recycling, and due to its low energy consumption and optimization of the reaction time of the sonochemical method [60]. The use and synthesis of MOFs has received attention in recent years. Most MOFs are synthesized by soluthermal (in which toxic solvents such as DMF, methanol are used) and hydrothermal (water solvent is used and require high energy consumption) methods [61], [62]. Sometimes, the characteristic and desired performance of MOF cannot be predicted during synthesis or before, so that there may be interference between different functional groups and the adsorbent structure is not made of desired ligands. It is also possible to destroy the ligands in the synthesis process. In this situation, using methods such as sonochemical synthesis or room temperature (in which solvents such as ethanol and water are used and consume less energy), an organic metal framework with desired properties can be achieved [63]. Considering the presence of pharmaceutical pollutants in drinking water and the side effects they have; this article examines the removal of the pharmaceutical pollutant azithromycin from contaminated water by a novel ZIF/zeolite composite adsorbent with high chemical stability in aqueous environments and high adsorption capacity. In this research, ZIF-8/zeolite composite was synthesized for the first time using the sonochemical green method. The effect of process factors, such as pH, pollutant concentration, adsorbent regeneration, kinetic models, adsorption isotherm and thermodynamic in the aqueous medium was studied.

## 2 Materials & methods

### 2.1 Materials and devices

Zeolite (CAS Number: 1318-02-1), Zinc nitrate hexahydrate, 2-Methylimidazole, Triethylamine, Ethanol, Methanol, Hydrochloric acid, and Sodium hydroxide were provided from Sigma-Aldrich with at least sincerity of 99%. Azithromycin drug was purchased from Farabi Pharmaceutical Co., Iran. Ultrasonic homogenizer of Banlin device, model HD-12207UW2200 made in Germany (frequency range: 20–500 kHz, time range: 0.01 to 99 min), and from Bio-Opic ultrasonic bath model USC2840-I Series made in China (Capacity: 0.6-45L, ultrasonic Freq: 40 kHz and time setting with 1–30 min, built-in heating up to 80 °C) was used for synthesis. XRD analysis was conducted with Advance D8, made in Germany, using Cu K α at 1.540598° A with Nickle at 2θ scan rate of 1° to 85°. The angle measurement precision was 0.001. FT/IR 6300 (JASCO, Japan) was used for FTIR analysis with a resolution of 4 cm^−1^. Furthermore, the MIRA III device from TESCAN Company (Czech Republic) was used for the SEM images. BELSORP MINI II instrument (Japan) was used for Brunauer-Emmett-Teller (BET) analysis. Azithromycin adsorption measurement was conducted with a spectrophotometer with DR-5000 (JASCO, Japan).

### 2.2 Preparation of ZIF-8/Zeolite composite

The method presented by Li et al. [64] was used for the synthesis of ZIF-8. All parameters related to using the ultrasonic bath were chosen based on the study of Lee et al. [64]. The findings of this study's comparison of various synthesis methods revealed that using ultrasonic waves reduces the size of the crystals, increasing the specific surface area. They studied the synthesis time and their results showed that the yield for 2 h and 4 h is close to each other, so 2 h was chosen for the synthesis.

Zeolite was washed with ethanol to remove the pollutants from its surface, and put within a vacuum oven for 24 h at 150 °C to activate its sites. For the synthesis of ZIF-8/Zeolite composite, 1.6 g of 2-methylimidazole were added to 50 ml of methanol in the round flask. 0.73 g of zinc nitrate hexahydrate was added to 50 ml of methanol. The solutions were stirred for 30 min and were added together. Then, they were stirred for 10 min. The sonicator used with an adjustable power output (maximum 500 W at 20 kHz) for 1 h. The solution was put within an ultrasonic bath at 60 °C for 1 h. The solution was centrifuged at 5 min at 10000 rpm. Hence, obtained gel was dried at 120 °C for 1 h in the oven. The white material was washed three times with ethanol for 48 h separated by centrifuge at 11000 rpm and 10 min. Finally, the powder was dried in a vacuum oven for 12 h at 80 °C.

**Fig. 1:**
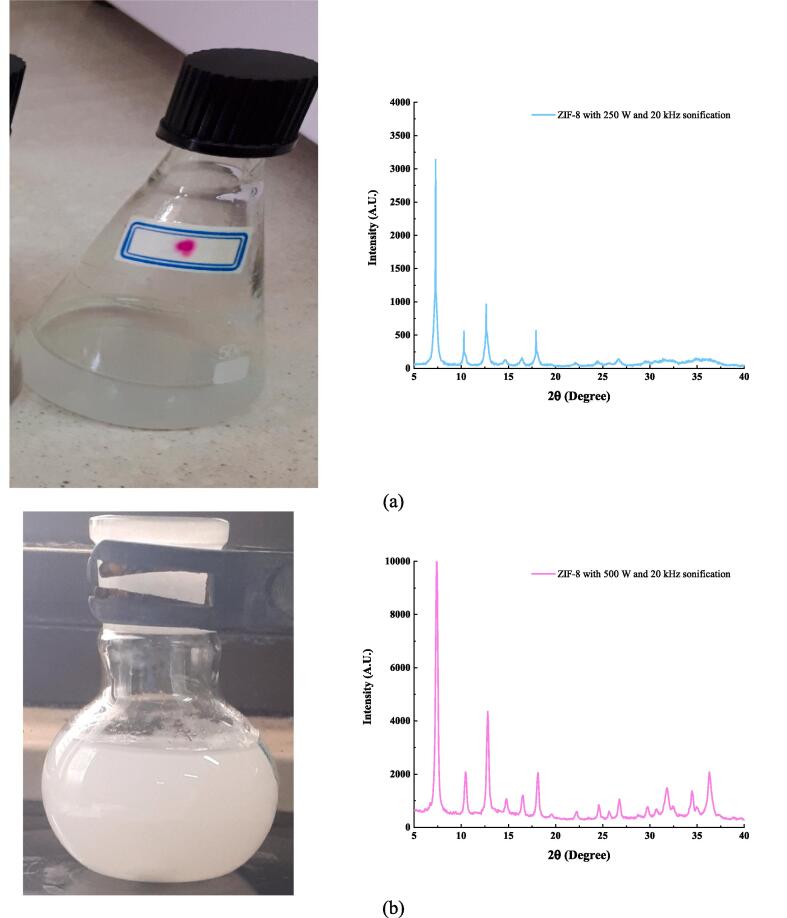

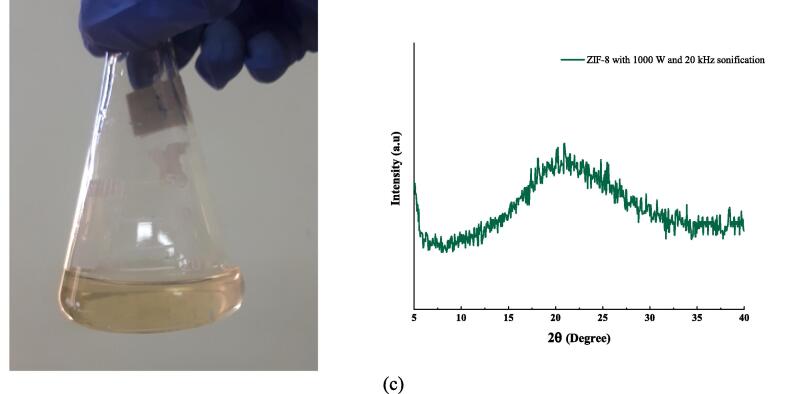
The synthesized materials with a) 250 W,20 kHz, b) 500 W,20 kHz, and c) 1000 W, 20 kHz sonification.

In this study, screening tests were conducted for the ultrasonic power of the homogenizer. First, the optimal conditions of Lee et al. [64], equal to 1 h, 500 W power and 20 kHz frequency, were performed. Then, two syntheses were performed under the same time conditions with the power of 1000 and 250 W and the frequency of 20 kHz. In these conditions, more power caused the degradation of ligands. Also, the amount of synthesized material in the condition of 250 W is very low and the efficiency of this method is 30%. Meanwhile, the synthesis efficiency for the conditions of 500 W and 20 kHz frequency is equal to 87%. Also, the XRD pattern was taken from all the samples, the results showed that we have the highest crystallinity in the optimum mode of 500 W and 25 kHz. Fig. 1 shows the synthesized materials in each of the conditions.

**Fig. 2:**
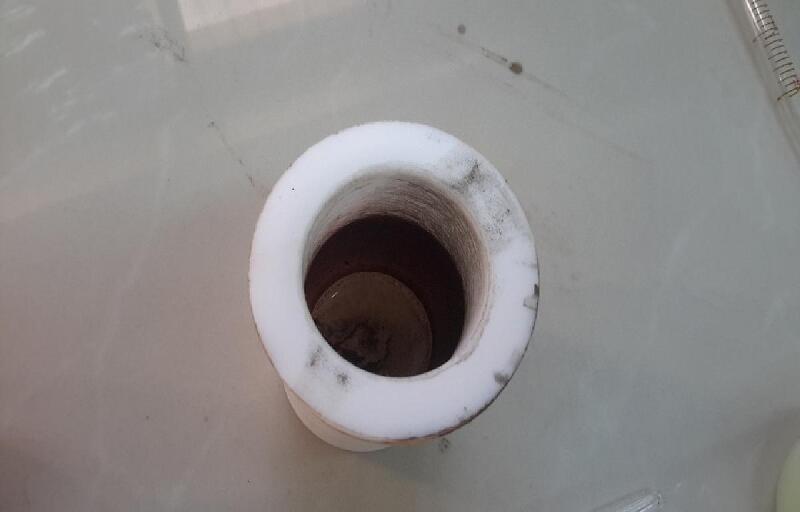
The results of solvothermal synthesis of ZIF-8/Zeolite.

According to the conducted research, the synthesis method is only effective on the size of nanoparticles and has no effect on the morphology of the particles, and room temperature and solvothermal methods were also used for composite synthesis, in the solvothermal method due to the high temperature and pressure This method is used, the material loses its properties and burns (Fig. 2).

Also, the room temperature method was also investigated, which had a low efficiency and the specific surface area of the ZIF-8/Zeolite in this method was lower (750 m^2^/gr) than the ultrasonic method. A lower specific surface area reduces the adsorption capacity, so the ultrasonic method was chosen.

### 2.3 Experimental method

For the adsorption isotherm experiment, 25 ml of a drug mixture comprising 50, 150, and 200 mg/l were combined with 0.025 g of adsorbent. Then, the solution and adsorbent were shaken at 180 rpm for 2 hr. For the kinetic experiment, a drug solution with a C_0_ of 200 mg/l was generated, and 0.025 g of each adsorbent were added to 25 ml of the drug solution at different periods (5 to 120 min). To determine appropriate pH for the adsorption of azithromycin, 0.025 g of adsorbent was included in 25 ml of drug solution with C_0_ = 200 mg/l, at pH 5–9. For the reusability experiment, 50 mg of adsorbent placed in 35 ml of azithromycin solution with C_0_ = 200 mg/l and pH = 8 at 25 °C for 2 h until the adsorption reached the equilibrium. Afterward, the adsorbent was separated using Sinter Glass 5G which was placed in Water/Ethanol (20:80) solution. All tests involving adsorption were conducted three times, and the results were recorded. There was an error bar in each figure that displayed the average error. The adsorbent was recycled 10 times, and the adsorption procedure was performed each time. After achieving equilibrium at each step, the drug solution was delivered to the cell using a micropipette, and absorbance was measured using a UV–Vis spectrophotometer. The adsorption loading is denoted by q, calculated from Eq. (1).

(1) $$q=\frac{\left(C_{0}-C_{e}\right)\times V}{m}$$

In Eq. (1), q is the adsorption loading (mg/g), *C_0_* is the primary concentration (mg/l), *C_e_* is the concentration of drug after the adsorption experiment (mg/l), V is the volume (l), and *m* is the adsorbent used in the experiment (g).

## 3 Results and discussion

### 3.1 Characterization

#### 3.1.1 XRD analysis

**Fig. 3:**
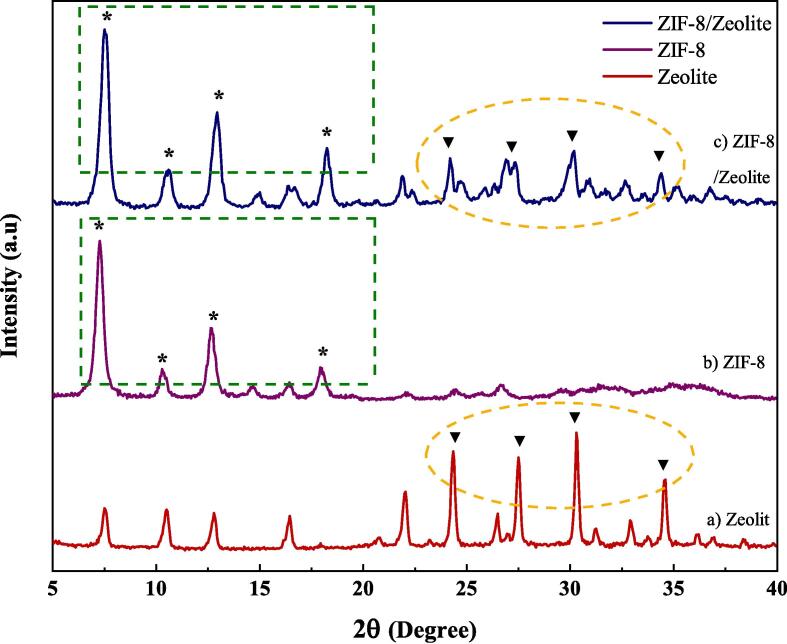
The XRD pattern of (a) purchased zeolite, (b) sonochemically synthesised ZIF-8, and (c) sonochemically synthesised ZIF-8/Zeolite.

Fig. 3(a) belonged to the XRD pattern for the purchased zeolite and showed a peek at 2θ of 22°, 24°, 27°, 30°, and 35°, which was compatible with the card numbers 39-0222 of Zeoltie A from the ICDD (International Centre for Diffraction Data) [65]. The crystal size obtained from the Scherrer equation was 33 nm. Fig. 3(b) shows the XRD pattern for the sonochemically synthesised ZIF-8, and observed peaks at 2θ of 7°, 12.5°, and 17°. Where as ZIF-8 was synthesized using Ref. [64]. The XRD results for the ZIF-8 were in accordance with the reference [64]. Moreover, based on Scherrer equation, the crystal size was 9.3 nm. Fig. 3 (c) shows the XRD pattern for the sonochemically synthesised ZIF-8/Zeolite. The peaks observed at 2θ of 7.5°, 13°, and 18.3° belonged to ZIF-8, and the peaks observed at 2θ of 24.2°, 27.3°, 30.15°, and 34.4° belonged to zeolite. Regarding the coverage of zeolite with ZIF-8, the intensity of peaks of zeolite decreased. The presence of peaks of both materials in the XRD analysis and their sharp intensity showed that composite materials were synthesized and that their crystallinity was high.

#### 3.1.2 FTIR analysis

**Fig. 4:**
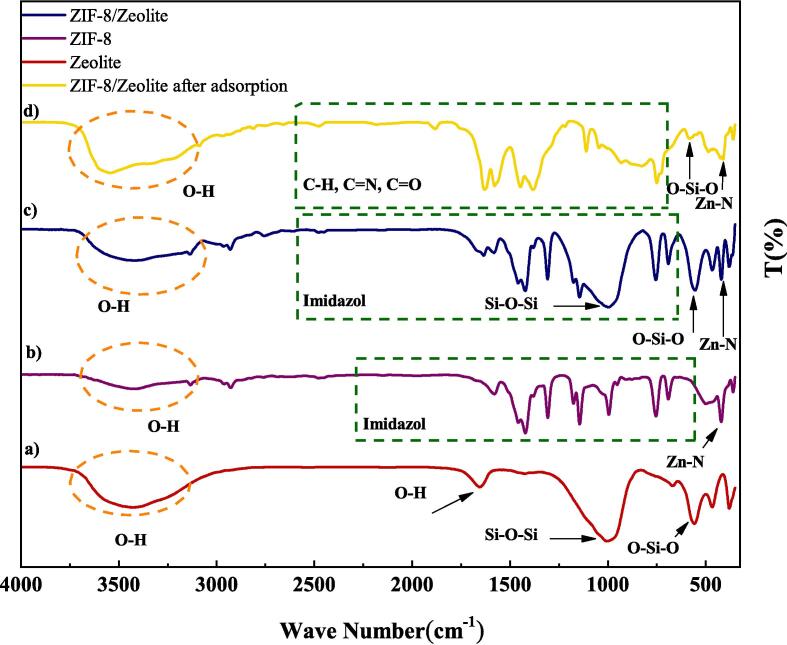
FTIR spectrum of (a) Zeolite, (b) ZIF-8, and (c) ZIF-8/Zeolite and d) ZIF-8/Zeolite after adsorption.

FTIR analysis was accomplished over 400–4000 cm^−1^, and the result is shown in Fig. 4. In Fig. 4 (a), FTIR for zeolite was observed. At 567 cm^−1,^ the vibration band of O-Si-O, at 1013.8 cm^−1^ vibration of Si-O-Si, and 1668 cm^−1^ and 3439 cm^−1^ vibration band of O-H were perceived. Fig. 4(b) is FTIR for synthesized ZIF-8. The peaks at 650 cm^−1^ belonged to the vibration band of C-H, and the band of 1500 cm^−1^ belonged to the vibration band of C = N in the imidazole ring, respectively. The stretching band of Zn-N was represented by the bands at 440 cm^−1^, which supported the band's development between a metal and an organic ligand. FTIR for composite is shown in Fig. 4(c). In these spectra, all bands of zeolite and ZIF-8 were observed, which confirmed the synthesis of composite. Fig. 4(d) shows the FTIR of composite sample after adsorption. Based on Fig. 4, after adsorption, hydrogen bonds were established between the adsorber and the pollutant. The presence of C-H and C = N bands was related to imidazole.

#### 3.1.3 BET analysis

**Fig. 5:**
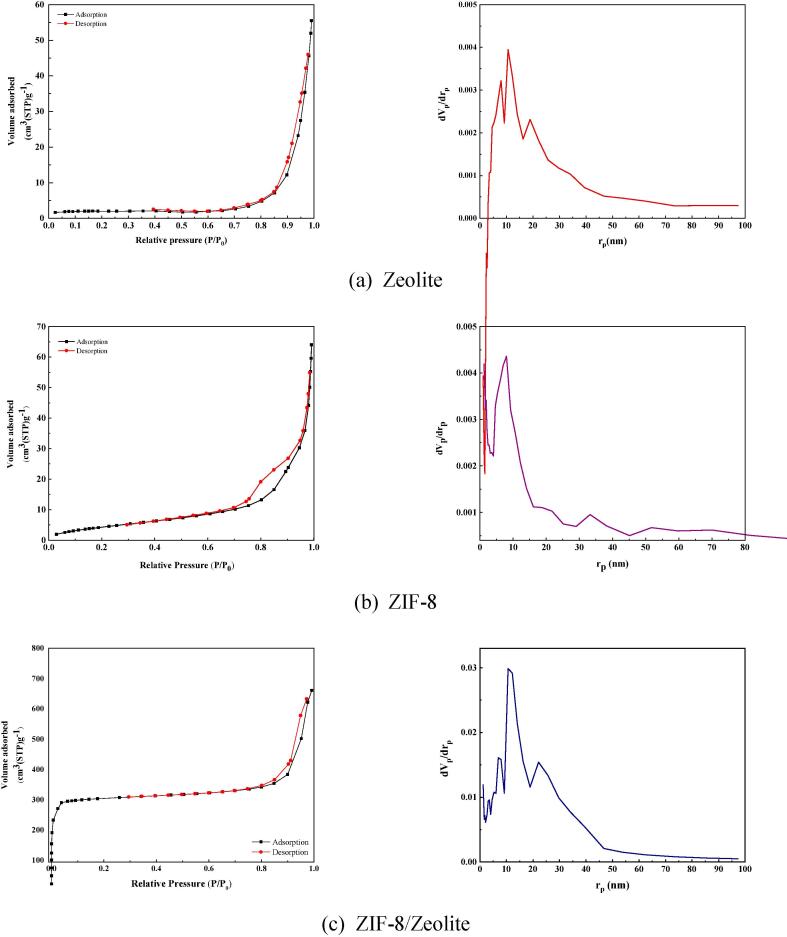
BET & BJH diagram of (a) Zeolite, (b) ZIF-8, and (c) ZIF-8/Zeolite.

Using the BET, surface area was calculated. Based on nitrogen gas adsorbed at relative pressures between 0.1 and 1, surface area was calculated. Fig. 5 displays adsorption and desorption isotherms as well as a BJH diagram. Due to the adsorption in mesopores at higher pressures, nitrogen adsorption in micropores took place at low partial pressures. Capillary condensation in mesopores led to the hysteresis. Pore size distribution was determined by the BJH method. The highest pore frequency for zeolite, ZIF-8, and composite had radius sizes of 10.63 and 1.21 nm, which were in the range of mesopores. Based on IUPAC [66], adsorption and desorption isotherms for zeolite, ZIF-8, and composite were similar to pseudotype II, type IV, and type 4 hysteresis, respectively. This difference was in terms of different pore sizes in composite, and the pores had a cylindrical morphology. The surface area for zeolite, ZIF-8, and composite is 8.89, 1123, and 887 m^2^/g. Table 1 shows the surface specification of adsorbent [67].

**Table 1:** The textural properties of Zeolite, ZIF-8, and ZIF-8/Zeolite.

parameter	Zeolite	ZIF-8	ZIF-8/Zeolite
**a_s_ (m^2^/g)**	8.89	1123	887.5
**V_m_ (cm^3^(STP) g^−1^)**	1.83	310	203.91
**V_p_ (cm^3^g^−1^)**	2.04	0.21	0.33
**r_p_ (nm)**	10.63	10.63	1.22

#### 3.1.4 SEM analysis

**Fig. 6:**
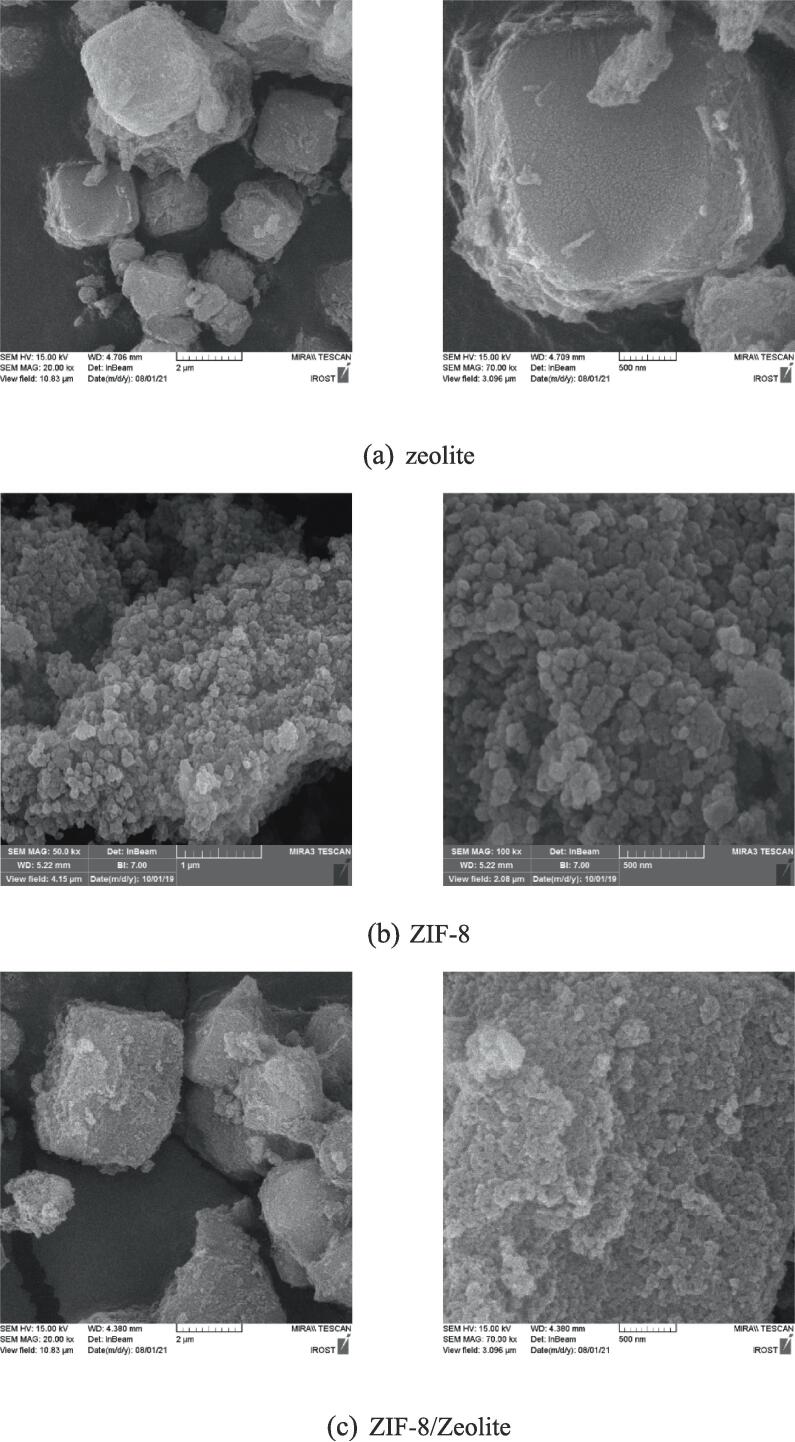
SEM pictures of (a) Zeolite, (b) ZIF-8, and (c) ZIF-8/Zeolite.

Fig. 6 shows the results of SEM analysis at 2 µm and 500 nm. Zeolite was multi-dimensional. Fig. 6(b) shows ZIF-8, which indicated that this material was multifaceted and synthesized correctly. Fig. 6(c) shows the ZIF-8/Zeolite composite. ZIF-8 coated the zeolite surface in terms of its nanometer size.

Regarding the input of ICP device should be a clear liquid or solution, therefore, to examine solid samples, it is necessary to dissolve the solid material in a suitable solvent, which is called the digestion process [68]. MOFs, such as ZIF-8 are dispersed and do not dissolve in aqueous and organic solvents, such as methanol and ethanol and dissolve only in acidic environments with a pH of less than 3, and zeolites only in alkaline environments. It dissolves with a pH higher than 10 [69], [70], [71]. Consequently, it is not possible to get an ICP test for the composite in the investigated pH range.

### 3.2 Adsorption tests

In the field of adsorption, researchers have investigated the effect of contact time (kinetic), temperature (thermodynamics) and initial concentration (isotherm) [72], [73]. In adsorption, temperature, concentration, contact time and adsorbent dose and pH affect the adsorption capacity. In this research, all parameters affecting the adsorption process have been investigated. Also, the stability and recovery of adsorbents have been investigated.

#### 3.2.1 Effect of dosage

**Fig. 7:**
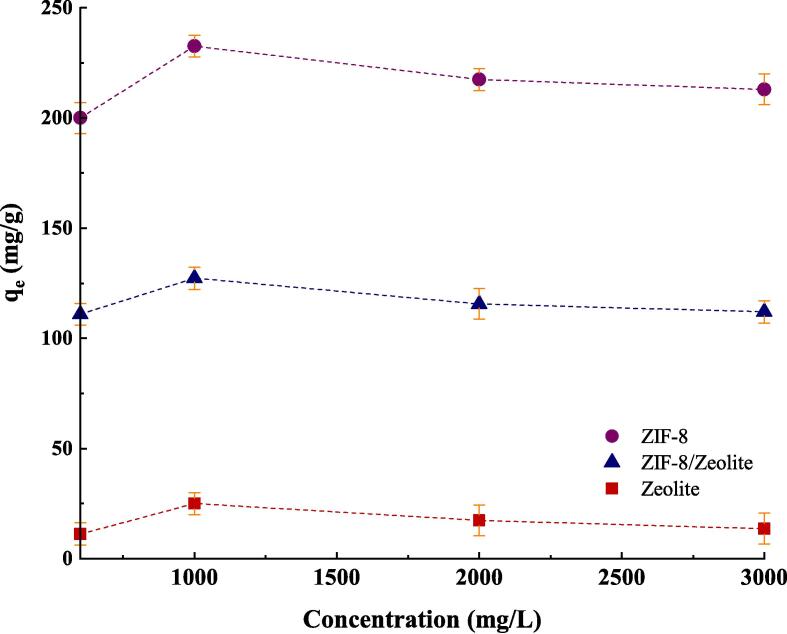
The adsorption capacity of azithromycin in terms of adsorbent dosage (C_0_ = 200 ppm, v = 25 ml, time = 120 min, T = 25 °C).

To check the adsorbent dose 5, 25, 50 and 75 mg of adsorbent are mixed in 25 ml of drug solution and then the amount of adsorption was measured. Fig. 7 shows the adsorption capacity of azithromaycine due to the adsorbent dosage. The researchers showed that by increasing the adsorbent dose, the adsorbent particles stick together and aggregation occurs. Small amount of adsorbent becomes saturated in the face of a large amount of pollutant [74]. During the experiments, the solution was stirred by an incubator shaker. If the amount of adsorbent is high, mixing the solution will cause the particles to gather in the middle of the container and stick together. On the other hand, if the solution is mixed with a stirrer and a magnet, the presence of a magnet causes the drug to not easily penetrate into the pores of the MOF and is known as a disturbing factor. The opinion of the respected referee for the low amount of adsorbent is completely correct. Due to the different specific levels, the saturation level values of the adsorbents are also different. But the drug concentration is so high that the difference is only in the time of saturation. The amount of 5 mg of each of the adsorbents is saturated in the first moments and the so-called adsorbent is poisoned. The saturation time for the ZIF-8, which has the highest specific surface, is 5 min. As a result, the amount of 25 mg was chosen.

#### 3.2.2 Effect of pH

**Fig. 8:**
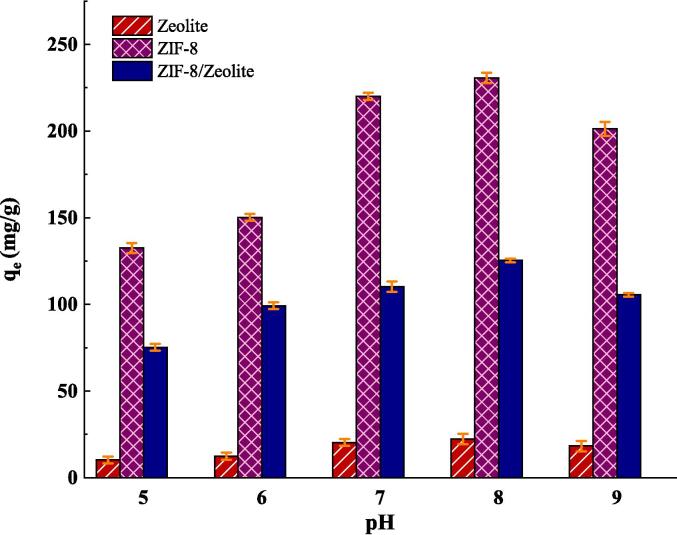
The effect of pH on the azithromycin adsorption on Zeolite, ZIF-8, and ZIF-8/Zeolite (c_0_ = 200 ppm, m = 0.025 g, v = 25 ml, time = 120 min, T = 25 °C).

When it comes to adsorption and electrostatic interaction, pH is an important parameter. The p*K*a of azithromycin was 8.5, and the isoelectric point of zeolite, ZIF-8, and composite were 7, 8.5, and 8, respectively [75]. The effect of pH on adsorption loading is shown in Fig. 8. Based on the surface charge of contaminant, ZIF-8, and composite, since at the isoelectric point, the p*K*a of the drug was 8.6, the adsorbent and contaminant had similar surface charges. At pH 8, both materials were neutral, and thus maximum adsorption occurred. By zeolite, the surface charge of the contaminant and the adsorbent was identical and positive at pH levels lower than 7, which decreased the adsorption capacity. However, the highest adsorption occurred from pH 7 to 8.5 in terms of the opposite charges of adsorbent and contaminant. For pH values higher than 8.5, due to similar surface charges, adsorption capacity decreased. PH 8 was selected as the optimum pH value [76], [77], [78], [79].

**Fig. 9:**
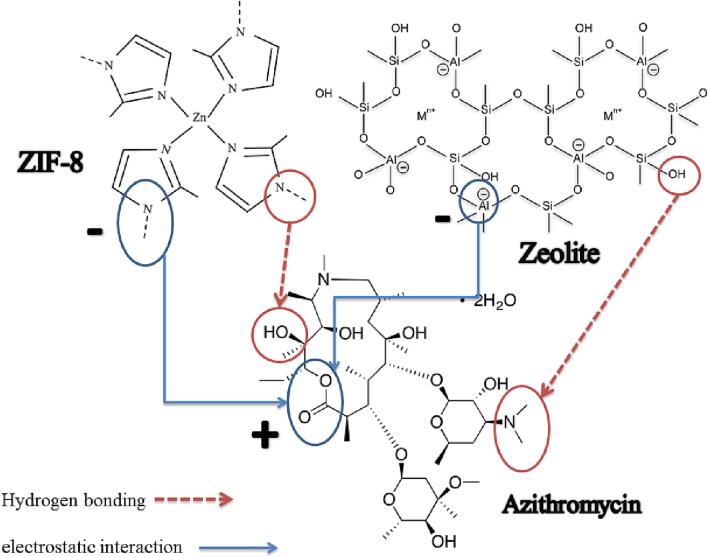
The mechanism of adsorption of Azithromycin on ZIF-8/Zeolite.

The mechanism by which azithromycin is adsorbed on ZIF-8/Zeolite is shown in Fig. 9. The mechanism of electrostatic interaction has an impact on the adsorption process when taking into account the effect of pH on the adsorption capacity. Moreover, based on the structure of drug and adsorbent, the presence of functional groups OH in the structure of zeolite, amino groups in the structure of ZIF-8, as well as the presence of acidic functional groups (–CH_3_, COOH) in the structure of drug, the acid-base interaction mechanism and hydrogen bonding were involved in the adsorption process [29], [31], [80], [81].

#### 3.2.3 Kinetic of adsorption

The kinetic tests were fulfilled to examine the effect of contact time on drug take-up and determine the equilibrium time. The data has been fitted with the relevant equations using the curve fitting tab of MATLAB version 2018. The information was adapted with pseudo-first-order, pseudo-second-order, and intraparticle diffusion models, and obscure quantities of kinetic models were obtained. Under Table 2 and R2, pseudo-second-order kinetic was an acceptable model for drug adsorption. This model assumed that the reaction was a rate-limiting reaction. According to a study of intraparticle diffusion, the constant penetration speed of intraparticles ranged from 2 to 19. The constant C indicated the boundary layer's thickness, which represented the outside mass transfer. According to the study, mass transfer on the outer surface controls the first stage of adsorption. On the contrary, the sharp increment in adsorption within the method's early stages was considered a quick stage for starting the mass transfer [24].

**Table 2:** Parameters of kinetics models.

Model	Parameter	Zeolite	ZIF-8	ZIF-8/Zeolite
**pseudo-first-order**	*q_e_ (mg.g^−1^)*	22.78	229.2	124.2
	*K_1_ (*min*^−1^)*	0.05	0.18	0.15
	*R^2^*	0.97	0.97	0.97
**pseudo-second-order**	*q_e_ (mg.g^−1^)*	26.19	231.2	126.5
	*K_2_/10^−2^ (g/(mg.*min*))*	0.26	0.72	0.62
	*R^2^*	0.98	0.98	0.98
	*R^2^adjust*	0.98	0.99	0.99
	*Residual Sum of Squares*	7.66731	2.00461	1.86837
	*Reduced Chi-Sqr*	1.91683	0.50115	0.46709
**Intraparticle diffusion**	*C (mg/g)*	3.17	62.98	32.75
	*k_ipd_ (mg.*min *^−0.5^ g^−1^*)	2.11	19.57	10.75
	*R^2^*	0.86	0.67	0.70

**Fig. 10:**
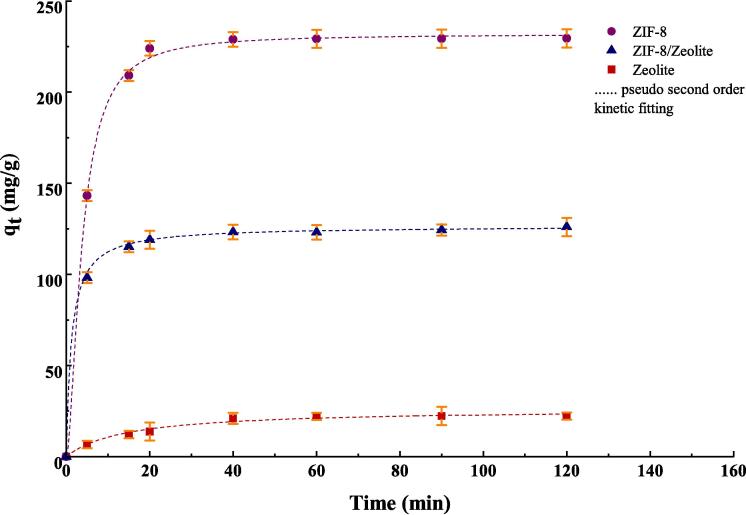
The pseudo-second-order kinetic fitting for azithromycin adsorption on the Zeolite, ZIF-8, ZIF-8/Zeolite (c_0_ = 200 ppm, m = 0.025 g, v = 25 ml, pH = 8, T = 25 °C).

Based on Fig. 10, in the initial stages, the adsorption speed was higher in terms of higher available sites on the adsorbent and higher C_0_ of pollutant (the slope of graph was sharp). By approaching the equilibrium, activation sites were occupied. Thus, the speed of adsorption decreased (the slope of graph slowed down and approached zero). The adsorption graph remained constant as long as the adsorbent could not adsorb more pollutants.

#### 3.2.4 Investigation of the adsorption isotherm

**Fig. 11:**
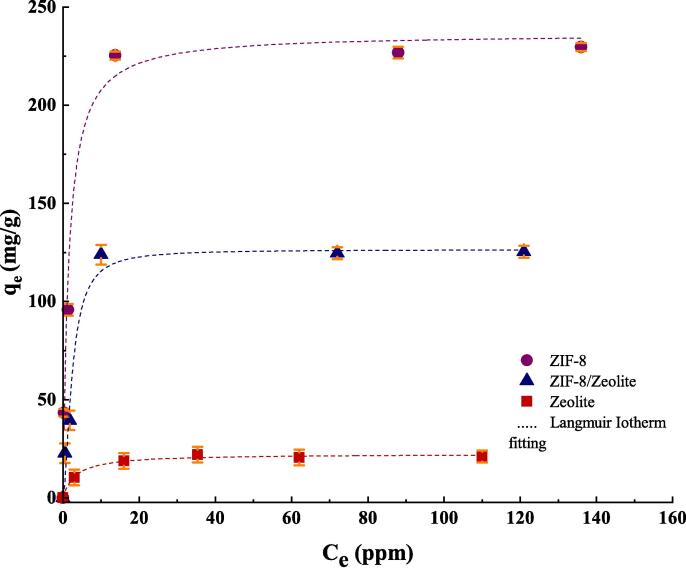
Langmuir isotherm fitting diagram (Time = 120 min, m = 0.025 g, v = 25 ml, pH = 8, T = 25 °C).

Fixed values of adsorption isotherm were determined to be specific to surface properties, and its fondness for adsorption prepare [82]. The isotherm models were studied, and the results are shown in Table 3. The q_m_ indicated the adsorption loading. The regression coefficient (R^2^) for ZIF-8, Zeolite, and ZIF-8/Zeolite in Langmuir model was 0.99. Consequently, the laboratory data were consistent with fitting data. *K_L_ *in Langmuir model was used to precisely determine the particle attraction power, and the amount changed from 0.32 to 0.62. The maximum adsorption loading for azithromycin was 235.3 mg/g. R^2^ for zeolite, ZIF-8, and ZIF-8/Zeolite were 0.89, 0.9, and 0.94, respectively, which were lower than Langmuir isotherm. This result confirmed the unsuitability of Freundlich isotherm for the adsorption of drug. K_F_ was determined to be 10.8, 102.3, and 45.3, respectively. The Freundlich isotherm parameter n indicated the suitability of adsorption. Regarding the information in Table 4, if n is from 0 to 10, the adsorption is favorable [83]. According to the results obtained for the adsorbent, the Langmuir model and the Redlich-Paterson model had the highest R^2^ compared to the other isotherm models. The adsorption of azithromycin on studied adsorbents was a monolayer. The adsorbent texture was homogenous and adsorption was physical and chemical. Regarding the proximity of β in the Redlich-Paterson model to the value of 1, Langmuir model had higher compatibility with experimental data. According to the specific surface area of the adsorbents, ZIF-8 is the highest and zeolite is the lowest, the highest and lowest adsorption capacity is related to ZIF-8 and zeolite, respectively. Fig. 11 shows the fitting of Langmuir model [84], [85]. According to the results of the investigation of the isotherms, the adsorption capacity also increased as the initial concentration increased. See Table 5..

**Table 3:** Adsorption isotherm parameters.

Model	Parameter	Zeolite	ZIF-8	ZIF-8/Zeolite
**Langmuir**	*q_max_ (mg.g^−1^)*	22.37	235.3	131
	*K_L_ (l.mg^−1^)*	0.305	0.625	0.3212
	*R^2^*	0.99	0.99	0.99
	*R^2^adjust*	0.98	0.98	0.98
	*Residual Sum of Squares*	3.50441	314.12864	749.06213
	*Reduced Chi-Sqr*	0.8761	157.06432	374.53107
**Freundlich**	*K_F_ (mg^1−n^l^n^.g^−1^)*	10.81	102.3	45.3
	*n*	6.23	5.56	4.352
	*R^2^*	0.94	0.89	0.90
**Temkin**	*B_T_ (Kj.mol^−1^)*	3.063	30.65	20.08
	*A_T_ (**L.mg**^−1^)*	16.36	23.56	6.863
	*R^2^*	0.81	0.89	0.92
**Redlich-Peterson**	*K_RP_ (**l.mg**^−1^)*	5.28	122.3	33.62
	*α_RP (_**_L.mg_**^−1^_)_*	0.17	0.43	0.18
	*β_Rp_*	1.075	1.043	1.073
	*R^2^*	0.99	0.99	0.99
**Hill**	*q_H_ (mg.g^−1^)*	21.46	234.1	128.5
	*K_H_*	4.85	1.621	3.14
	*n_H_*	1.36	1.04	1.137
	*R^2^*	0.99	0.99	0.99

**Table 4:** The comparison of the results of this study with the results of researchers.

Drug	Adsorbent	Adsorption capacity (mg/g)	Time	Isotherm	Ref.
Azithromycin	Waste-Product-Derived Graphene Oxide	55.5	15 min	Freundlich	[89]
	Activated carbon	41.84	120 min	Langmuir	[90]
	Magnetic activated carbon	42.38			
	Saponin-modified nano diatomite	91.7	60 min	Langmuir	[77]
	saponin-raw nano diatomite	68			
	ZIF-8	235.37	60 min	Langmuir	This study
	Zeolite	22.3			
	ZIF-8/Zeolite	131

**Table 5:** Thermodynamic parameters of the adsorption process.

Parameter					
Adsorbents	Δ*S (J/mol/K)*	Δ*H (J/mol)*	Δ*G (KJ/mol)*		
			Temperature (K)		
			298	333	373
Zeolite	17.37	1333.98	−3.85	−4.42	−5.15
ZIF-8	2.33	559.40	−0.13	−0.20	−0.31
ZIF-8/Zeolite	5.48	754.85	−0.87	−1.08	−1.20

Considering the importance of accurate, healthy, and cost-effective synthesis of nanostructured adsorbents, an easy, green, and energy-free method at room temperature called green synthesis of MOFs has been of interest in the last few decades [86]. In general, it can be concluded that the amount of solvent used for the synthesis and activation or purification of MOF should be as low as possible, and also optimizing the reaction time is very important to reduce energy consumption [87]. The best desired solvent for the synthesis of MOFs is water and ethanol in terms of its harmless properties and present technologies for purification and recycling, and due to its low energy consumption, and the optimization of the reaction time of sonochemical method [59], [88]. It was mentioned that this composite is better synthesised than other adsorbents. In addition, the results showed that the composite synthesized was suitable for the removal of azithromycin, since azithromycin had a high adsorption capacity. Table 4 shows comparing the results of this study with the results of researchers.

### 3.3 Thermodynamic of adsorption

**Fig. 12:**
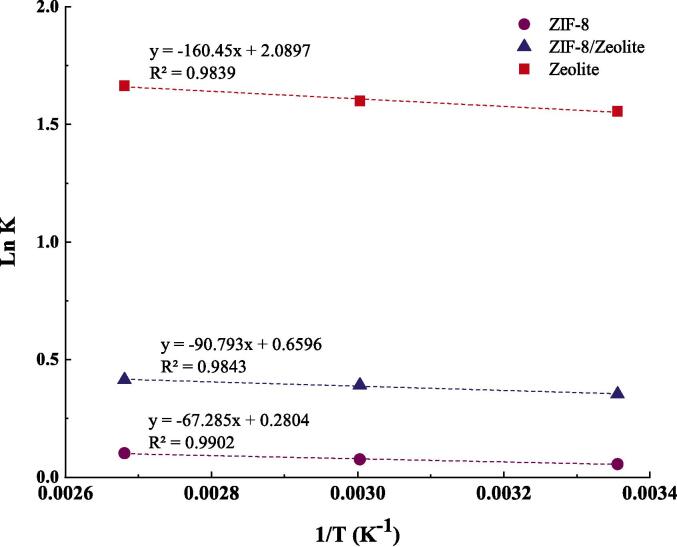
Van't Hoff curve to calculate enthalpy and entropy of adsorption.

Enthalpy and entropy may be calculated from the inclination and breadth of the Van't Hoff curve by researching how temperature affects adsorption capacity (Fig. 12) [91].

Table 4 lists the outcomes of the adsorption thermodynamics drawn from the Van't Hoff diagram. Table 4 shows that ΔG° was negative, demonstrating the spontaneity of the adsorption process. Throughout the procedure, irregularities in terms of the positive values of ΔS° increased. Based on the value of $\Delta H^{{}^{\circ}}$, which was positive, the adsorption process by all adsorbents was endothermic. Moreover, based on the references, and values of $\Delta H^{{}^{\circ}}$, which were elder than −20 kJ, it can be concluded that the adsorption band was physical [92], [93].

**Fig. 13:**
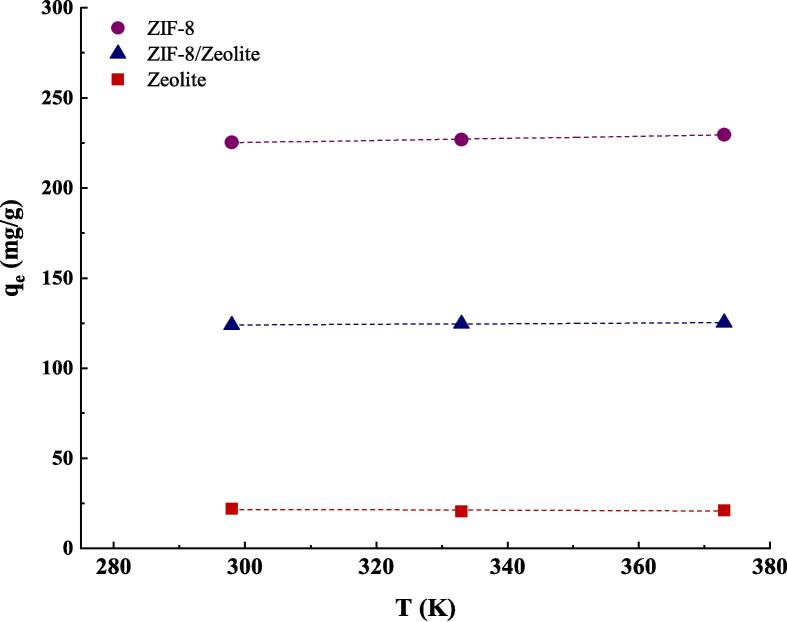
The changes in adsorption capacity based on the temperature.

Fig. 13 shows the changes in adsorption capacity based on the temperature. Adsorption increased with increase in temperature in physisorption. But, this increase in temperature does not always increase the adsorption capacity [94]. According to the adsorption theory, adsorption decreased with an increase in temperature and molecules adsorbed earlier on a surface tend to desorb from the surface at elevated[95]. Consequently, the temperature should be optimized. According to Fig. 13, by increasing the temperature, the adsorption capacity increased from 25 to 60 °C, but this increase is insignificant, and in terms of saving energy, the experiments were carried out at 25 °C.

### 3.4 Adsorbent regeneration

**Fig. 14:**
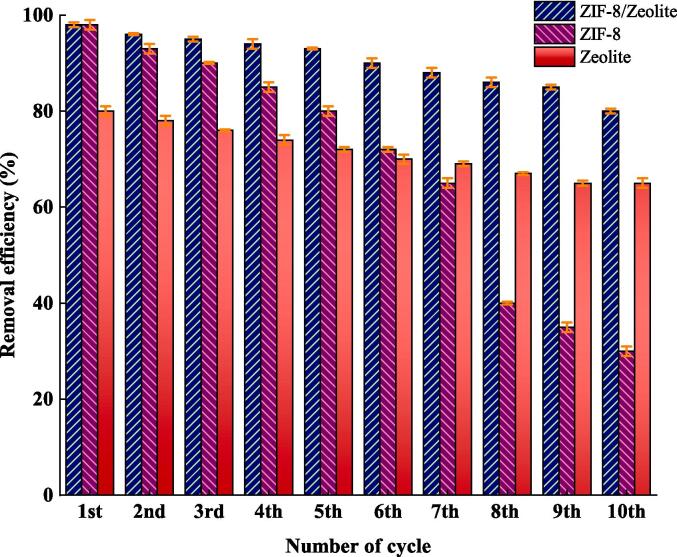
The number of cycles of azithromycin recovery on the zeolite, ZIF-8, ZIF-8/Zeolite.

**Fig. 15:**
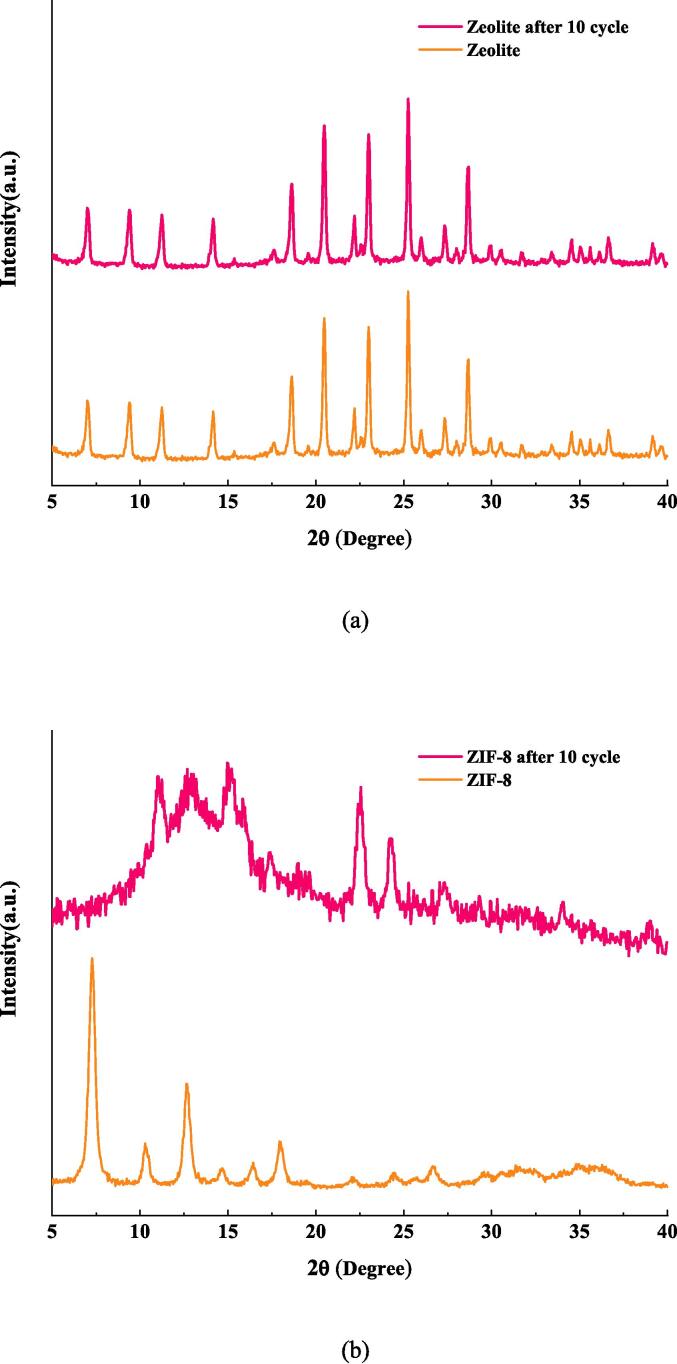

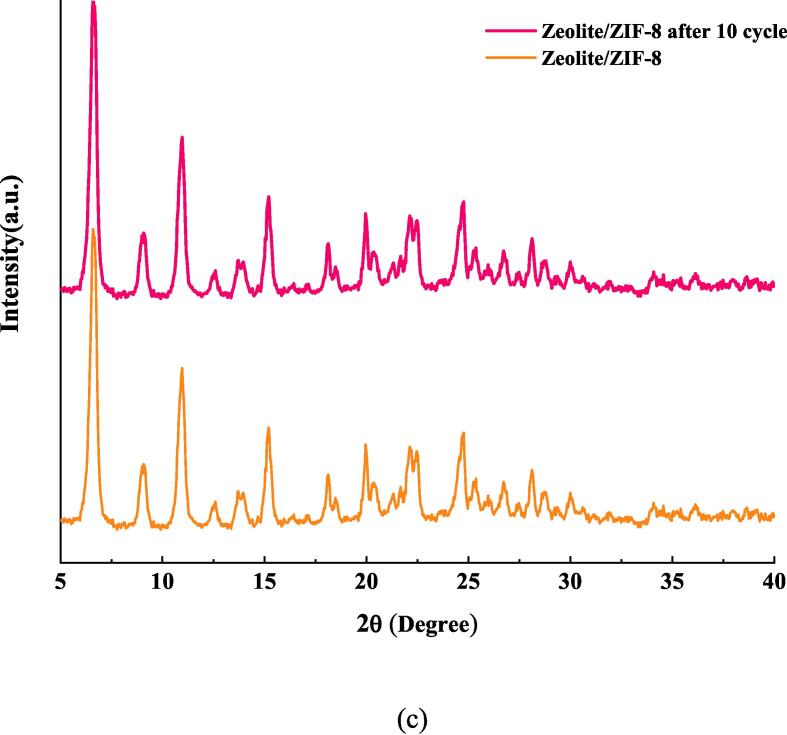
The XRD pattern of the a) zeolite, b) ZIF-8, c) ZIF-8/Zeolite after 10 cycle.

The recovery of the adsorbent was essential for reusing the adsorbent. As shown in Fig. 14, Zeolite, ZIF-8, and composite can be used in 10, 5, and 10 cycles in aqueous solutions. A lesser amount of composite was needed for higher removal of pollutants from aqueous solutions compared to zeolite. Based on Fig. 14, the removal efficiency of the composite was high enough during 10 cycles of usage. It indicated the stability of composite compared to ZIF-8. The adsorption capacity of composite was lower than ZIF-8, but composite was used in 10 recovery cycles and ZIF was used in 3 recovery cycles with a removal efficiency of 85%. Consequently, the composite was stable, and for a small amount of adsorbent, a larger amount of pollutants can be removed which was economical. Fig. 15 showes the XRD pattern of the zeolite, ZIF-8, ZIF-8/Zeolite after 10 cycle. According to Fig. 15, the synthesized composite was stable after 10 cycles and the XRD pattern did not change.

## 4 Conclusion

According to this study, the adsorption capacity on zeolite, ZIF-8, and composite was 22.37, 235.3, and 131 mg/g, and the data followed the Langmuir isotherm, which was a monolayer adsorption. The kinetic studies show that the drug adsorption obeyed pseudo-second-order kinetics, and the adsorbent reached equilibrium within 60 min, and at pH = 8. pH had a profound effect on the adsorption result due to the electrostatic interactions. The adsorption process was spontaneous, endothermic, which was associated with increased entropy. The recovery of adsorbent showed that the composite can be used in 10 cycles with a removal efficiency of 85%. The composite was stable in an aqueous solution, determined by the number of cycles used in the adsorption and reuse compared to ZIF-8.

## CRediT authorship contribution statement

**Zhiming Liu:** Methodology, Software, Validation, Writing – original draft, Investigation. **Ashkan Bahadoran:** Methodology, Software, Validation, Writing – original draft, Investigation. **As'ad Alizadeh:** Methodology, Software, Validation, Writing – original draft, Investigation. **Nafiseh Emami:** Methodology, Software, Validation, Writing – original draft, Investigation. **Tariq J. Al-Musaw:** Methodology, Software, Validation, Writing – original draft, Investigation. **Ahmed Hussien Radie Alawadi:** Methodology, Software, Validation, Writing – original draft, Investigation. **Aseel M. Aljeboree:** Methodology, Software, Validation, Writing – original draft, Investigation. **Mahmoud Shamsborhan:** Methodology, Software, Validation, Writing – original draft, Investigation. **Iman Najafipour:** Methodology, Software, Validation, Writing – original draft. **Seyed Erfan Mousavi:** Methodology, Software, Validation, Writing – original draft. **Milad Mosallanezhad:** Methodology, Software, Validation, Writing – original draft. **D. Toghraie:** Methodology, Software, Validation, Writing – original draft.

## Declaration of Competing Interest

The authors declare that they have no known competing financial interests or personal relationships that could have appeared to influence the work reported in this paper.

## Data Availability

No data was used for the research described in the article.
